# Metabolic reprogramming as an emerging mechanism of resistance to endocrine therapies in prostate cancer

**DOI:** 10.20517/cdr.2020.54

**Published:** 2021-03-19

**Authors:** Paolo Chetta, Giorgia Zadra

**Affiliations:** ^1^University of Milan, Milan 20122, Italy.; ^2^Current Address: Boehringer Ingelheim RCV GmbH & Co. KG, Dr.-Boehringer-Gasse 5-11, Vienna 1121, Austria.; ^3^Department of Oncologic Pathology, Dana-Farber Cancer Institute and Brigham Women’s Hospital, Harvard Medical School, Boston, MA 02215, USA.; ^4^Current Address: Institute of Molecular Genetics, National Research Council, Pavia 27100, Italy.

**Keywords:** Metabolic reprogramming, prostate cancer, castration resistance, endocrine therapies, metabolomics, metabolic imaging, therapy respo nse

## Abstract

Prostate cancer (PCa) is the second leading cause of cancer-related death in the US. Androgen receptor (AR) signaling is the driver of both PCa development and progression and, thus, the major target of current in-use therapies. However, despite the survival benefit of second-generation inhibitors of AR signaling in the metastatic setting, resistance mechanisms inevitably occur. Thus, novel strategies are required to circumvent resistance occurrence and thereby to improve PCa survival. Among the key cellular processes that are regulated by androgens, metabolic reprogramming stands out because of its intricate links with cancer cell biology. In this review, we discuss how cancer metabolism and lipid metabolism in particular are regulated by androgens and contribute to the acquisition of resistance to endocrine therapy. We describe the interplay between genetic alterations, metabolic vulnerabilities and castration resistance. Since PCa cells adapt their metabolism to excess nutrient supply to promote cancer progression, we review our current knowledge on the association between diet/obesity and resistance to anti-androgen therapies. We briefly describe the metabolic symbiosis between PCa cells and tumor microenvironment and how this crosstalk might contribute to PCa progression. We discuss how tackling PCa metabolic vulnerabilities represents a potential approach of synthetic lethality to endocrine therapies. Finally, we describe how the continuous advances in analytical technologies and metabolic imaging have led to the identification of potential new prognostic and predictive biomarkers, and non-invasive approaches to monitor therapy response.

## Introduction

Prostate cancer (PCa) is the most commonly diagnosed cancer and the second leading cause of cancer death in the US^[1]^. The unique dependency of PCa cells on androgen/androgen receptor (AR) signaling for their growth and survival is the basis for androgen deprivation therapy (ADT) as the first-line treatment in advanced disease. While ADT is initially effective, patients will eventually relapse within 18-24 months with a more aggressive form of the disease known as metastatic, castration-resistant prostate cancer (mCRPC), which is still incurable^[2]^.

Despite ADT-induced castrate levels of circulating androgens, the AR signaling is still functional in CRPC, and AR remains the major target of ongoing therapeutic efforts. Currently in-use second-generation AR signaling inhibitors, which include the FDA-approved AR antagonist enzalutamide and the steroid synthesis inhibitor abiraterone acetate, have significantly improved CRPC patient survival and quality of life^[2,3]^. However, their benefit is not long-lasting due to the occurrence of resistance mechanisms. These include AR gene/enhancer amplification, AR mutations leading to promiscuity, androgen-independent AR activation, coactivator overexpression, intratumoral *de novo* androgen synthesis, and overexpression of AR variants (AR-Vs), in particular AR-V7^[4]^. Expression of AR-V7, which can now be reliably measured in circulating tumor cells, has become a critical biomarker for therapeutic decision-making^[5]^. AR degradation through proteolysis targeting chimeras (PROTAC) (i.e., ARCC-4, ARD-61, ARD-69 and ARV-101) is currently under investigation to overcome mechanisms of castration resistance (CR)^[6-9]^. While this approach is showing promising results, the downside of PROTAC-based therapy is the potential risk of developing neuroendocrine-like PCas, which are a very aggressive and fatal subtype.

While acquired knowledge on AR-mediated mechanisms of resistance has driven the design of AR-focused, more effective treatments, it has also raised awareness that AR-indirect and/or independent mechanisms may contribute to CRPC. In this context, evidence for PCa metabolic heterogeneity paralleling genomic and transcriptomic heterogeneity and the role of metabolic reprogramming in the acquisition of CRPC features have started to emerge^[10,11]^.

In this review, we describe how androgens modulate cancer metabolism and lipid metabolism, in particular, and how their deregulation sustains resistance to endocrine therapies. We explore the interplay between oncogenes, tumor microenvironment (TME), and cancer metabolism in promoting disease progression and CR. We then discuss the potential of targeting metabolic vulnerabilities to overcome resistance to endocrine therapies and the role that diet/obesity may play in PCa progression^[12-15]^. Finally, we provide a contemporary perspective on the emerging role of metabolomics and state-of-the-art metabolic imaging in identifying prognostic and predictive biomarkers. The goal of this review is to call attention on the role of metabolic reprogramming in CR, and on the need for carefully designed clinical trials to evaluate metabolic interventions alongside endocrine therapies.

## Androgens and prostate cancer metabolic reprogramming

### Androgens and the rewiring of lipid metabolism in PCa

**Figure 1:**
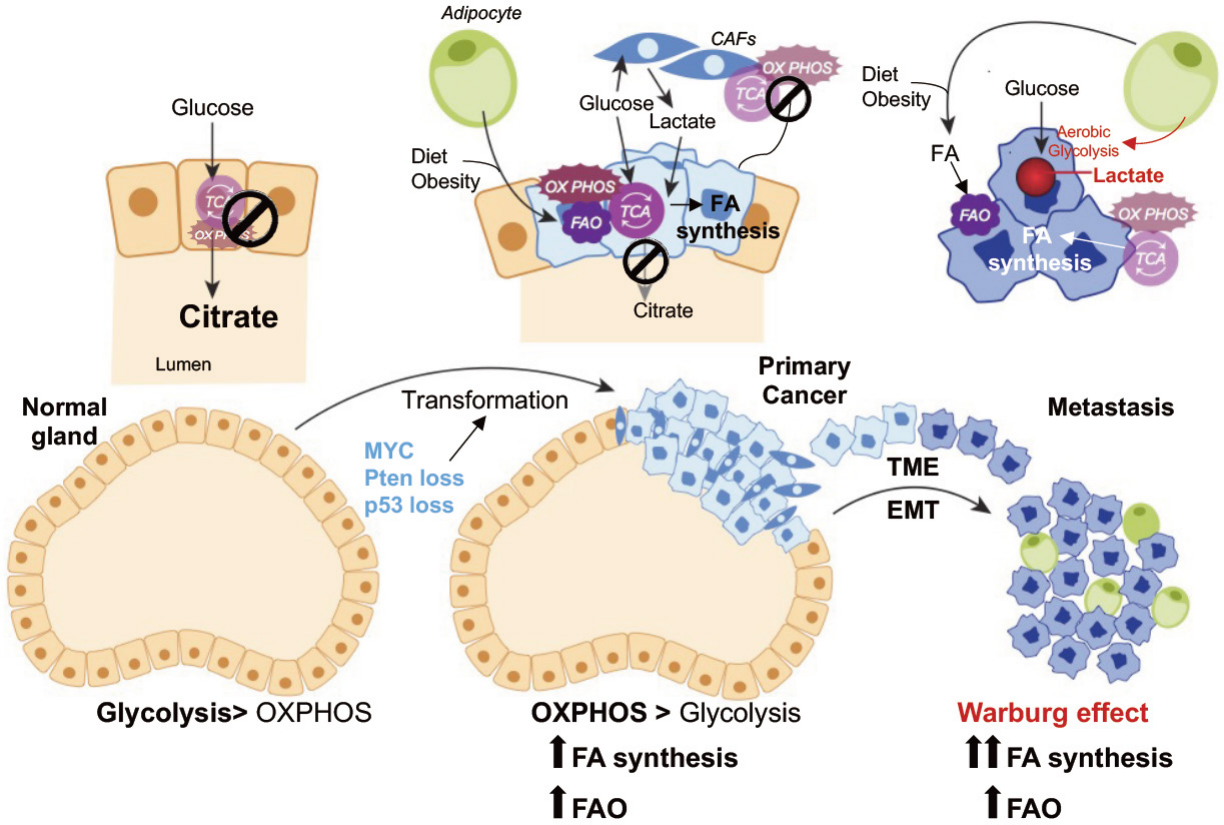
Metabolic alterations during PCa progression. Diagramatic representation of putative metabolic changes during progression from non-neoplastic disease to mCRPC. In normal prostate epithelial cells, the tricarboxylic acid cycle (TCA) cycle is truncated, and low rate of oxidative phosphorylation (OXPHOS) are observed. High levels of citrate are released in the seminal liquid. During malignant transformation, which is induced by genetic alterations (i.e., Pten loss, p53 loss, MYC overexpression), PCa cells reactivate the TCA cycle to oxidize citrate for energy production and convert citrate to acetyl-CoA for de-novo lipid synthesis. Fatty acids (FAs) from diet or obesity-associated adipocyte lipolysis also feed into the TCA cycle for energy production. Metabolic crosstalk is observed between cancer-associated fibroblasts (CAFs) and PCa cells, whereby CAFs provide lactate as fuel source. Increased aerobic glycolysis or the Warburg effect is observed in the metastatic stages of the disease. The Warburg effect seems to be stimulated by adipocytes in the bone marrow (BM). BM adipocytes promote the expression of glycolytic enzymes in mCRPC cells. Increased lactate secretion in TME is associated with tumor aggressiveness and metastases formation. Both *de-novo* lipogenesis and FAO are observed in the metastatic niche to fulfill the energetic and anabolic needs of mCRPC cells. EMT: epithelial-mesenchymal transition; TME: tumor microenvironment; PCa: prostate cancer; mCRPC: metastatic, castration-resistant prostate cancer

Androgens regulate cellular homeostasis, tissue differentiation, and maintenance of secretory functions of normal prostate cells. In PCa, however, the androgen/AR axis drives PCa cell growth and survival through the transcriptional regulation of many cellular processes, including cellular metabolism^[16]^. The latter shows significantly different features between normal and PCa cells. Normal prostate cells accumulate zinc, which inhibits the enzyme m-aconitase required for the conversion of citrate to isocitrate in the tricarboxylic acid (TCA) cycle. The process is sustained by androgens that favor zinc uptake and results in a truncated TCA cycle with secretion of a high quantity of citrate, a major component of the prostatic fluid. In PCa cells, zinc is no longer accumulated, and citrate can be oxidized via TCA cycle to produce energy and anabolic substrates, including acetyl-CoA for *de novo* lipogenesis^[17]^ [Figure 1]. Thus, PCa cells undergo a switch from citrate-producing to citrate-oxidizing cells. In 2011, the group led by Mills integrated chip-seq studies with transcriptomic and metabolomic profiling, and identified an anabolic transcriptional network involving AR as the core regulator. In this AR-modulated network, lipid metabolism stood out as a PCa hallmark^[18]^. Androgens regulate lipid metabolism mostly by promoting the expression and activation of sterol regulatory element-binding proteins (SREBPs), a family of transcription factors that bind to the promoter regions of genes involved in the synthesis, uptake and transport of fatty acids (FAs)^[19]^. Direct AR-binding sites have also been identified in the promoter region of FA synthase (FASN) gene^[20]^, and a recent genome-wide analysis has suggested that AR direct regulation may extend to many other lipogenic enzymes^[21]^. Thus, a combination of direct and indirect mechanisms is likely to be involved in AR-mediated control of FA metabolism.

Overexpression of lipogenic enzymes and accumulation of lipids has been documented in PCa cell lines, animal models, and human tissues using different methodologies, including biochemical approaches, radiolabeled tracers, nuclear magnetic resonance spectroscopy, histological staining, Raman spectroscopy and mass spectrometry (MS) imaging (MSI)^[22]^. Genetic/pharmacological perturbation of the AR axis confirmed androgen-regulation of lipid synthesis, largely via increased synthesis of FAs and cholesterol^[23]^. The overexpression of lipogenic enzymes favors the production of lipids as building blocks for new membrane synthesis, fuel, storage, intracellular signaling and post-translational modifications of oncogenic proteins, and it provides the maintenance of redox balance and response to DNA damage^[24-26]^, sustaining the fitness of cancer cells and their survival in a hostile microenvironment characterized by hypoxia and limited vascularity^[27]^.

Following this initial observation, androgens have been reported to stimulate the expression of a multitude of enzymes involved in lipid synthesis, binding, uptake, transport and oxidation, thereby influencing the entire lipid profile of PCa cells in a fine-tuned manner^[28,29]^. The works of Watt *et al*.^[30]^, Tousignant *et al*.^[31]^ and Schlaepfer *et al*.^[32]^ were particularly instrumental in elucidating the role of androgen-mediated FA uptake and FA oxidation (FAO) in both PCa development and progression, with high expression of androgen-modulated FA transporters in the metastatic setting^[30,31]^. In this context, it has also emerged that there is crosstalk between FA uptake, FAO and *de novo* lipogenesis, where a synergistic antitumor effect is achieved by their concurrent targeting^[30,32]^. Moreover, Itkonen and coworkers identified enoyl-CoA-isomerase 2 (ECl2), a novel AR target involved in FAO, which was found overexpressed in PCa samples and associated with poor outcome^[33]^.

These findings suggest a scenario where lipid metabolism in PCa is highly dynamic. It is likely that AR coordinates its actions with other transcription factors or oncogenes to establish or fine-tune specific metabolic programs. A better understanding of the androgen-mediated modulation of lipid synthesis/oxidation cooperation is crucial in the design of effective targeted therapies.

It is possible that *de novo* lipogenesis supports PCa development providing building blocks for membrane synthesis and signaling. Once tumors outgrow their vasculature network, oxygen and nutrients accessibility is compromised and PCa cells may favor FAO to support their viability. After dissemination, when oxygen and nutrients may again be available in the metastatic niche, PCa cells may adapt again their metabolism to favor aerobic glycolysis^[34,35]^, while FAO and *de novo* lipogenesis still remain highly active [Figure 1]. Although several speculations and preliminary observations have been made, clear experimental evidence to support this sequential metabolic rewiring is still lacking. Thus, a better understanding of this metabolic plasticity is key to prevent disease progression, as discussed below. Close scrutiny of publicly available human datasets has confirmed the significant increase in the expression of multiple genes involved in FA synthesis [i.e., ATP citrate lyase (ACLY), acetyl-CoA carboxylase and FASN], FA desaturation [i.e., stearoyl-CoA desaturase 1], FA elongation [i.e., very-long chain FA elongases (ELOVL5, 7)], FAO [i.e., carnitine palmitoyltransferase 1 (CPT1) and ECl2], and cholesterol synthesis [i.e., 3-hydroxy-3-methylglutaryl-CoA reductase (HMGCR)] in PCa compared to nonmalignant tissue, especially in the metastatic setting^[22]^.

### *De novo* lipogenesis, FAO and castration resistance

In 2004, Ettinger and coworkers for the first time observed increased expression of FA transcriptional modulators and lipogenic enzymes in the transition from hormone-sensitive to androgen-independent PCa^[36]^. Thereafter, the overexpression of lipogenic enzymes in mCRPC has been documented using several human PCa datasets, especially in mCRPC overexpressing AR-V7 and resistant to treatment with AR signaling inhibitors (enzalutamide and/or abiraterone). FASN was among the top ten genes overexpressed in AR-V7-high mCRPC, suggesting an interplay between AR-V7 expression and lipid metabolism deregulation^[37]^. This was confirmed by AR cistrome analysis showing AR-mediated reactivation of FA synthesis during the progression to CRPC and the involvement of AR-V7^[38]^.

**Figure 2:**
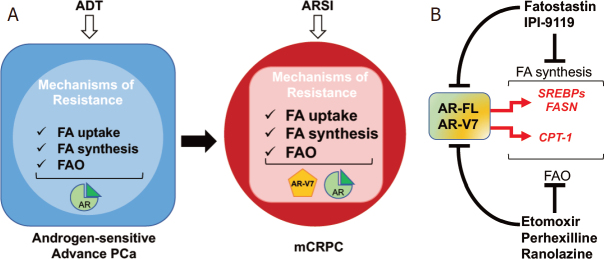
Metabolic mechanisms of resistance to endocrine therapies: the androgen receptor (AR) and lipid metabolism crosstalk. A: overview of lipid metabolism changes mediating resistance to androgen deprivation therapy (ADT) and androgen receptor signaling inhibitors (ARSI); B: schematic representation of AR signaling and lipid metabolism crosstalk. Drug targeting fatty acid (FA) synthesis and FA oxidation (FAO) are indicated as potential non-canonical approaches to inhibit AR signaling in mCRPC. CPT1: carnitine palmitoyltransferase 1; FASN: fatty acid synthase; SREBPs: sterol regulatory-element binding proteins

In 2019, the role for FAO in mediating CR also emerged. Joshi *et al*.^[39]^ demonstrated the involvement of CPT1, the rate-limiting enzyme for FAO initiation, in CRPC onset and anti-androgen resistance through histone acetylation. These new findings have opened a scenario, still not completely characterized, where both FA synthesis and oxidation contribute to CR in a fine-tuned manner^[40]^ [Figure 2A].

### AR and lipid metabolism mutual link in mCRPC

The original idea for a one-way regulation of lipid metabolism by androgens has been profoundly revised when groups including ours were able to achieve AR full-length (AR-FL) and AR-V7 downregulation following genetic and/or pharmacological inhibition of key lipogenic enzymes and transcription factors (i.e., HMGCR, SREBP and FASN), suggesting the existence of a mutual regulation between AR and *de novo* lipid synthesis^[41-43]^. Likewise, Schlaepfer *et al*.^[32]^ reported a significant decrease in AR-FL and AR-V7 as a result of FAO inhibition, highlighting for the first time the existence of a feed-forward loop also between FAO and AR signaling. These new findings have shifted the clinical perspective of targeting lipid metabolism in CRPC. Inhibitors of lipid metabolism are no longer merely repressors of an effector arm of AR oncogenic function, but they now offer a non-canonical approach to inhibit AR signaling and potentially overcome resistance to current standard of care, as we discuss below [Figure 2B].

### The emergence of a Warburg phenotype in aggressive PCa and mCRPC

In 1920, the German biochemist Otto Warburg reported a marked increase in aerobic glycolysis whereby cancer cells increase consumption of glucose and its conversion to lactate even in the presence of oxygen^[44]^ (a phenomenon known as the Warburg effect). The use of aerobic glycolysis, which is energetically unfavorable, provides cancer cells with anabolic precursors for a rapid proliferation while still fulfilling energy demand, and thus providing a selective advantage to adapt and thrive in harsh environments. Moreover, lactate, once considered merely a waste product, is now considered a key oncometabolite involved in angiogenesis, migration/invasion, metastasis formation, immune evasion, and drug resistance^[45]^. The discovery of a Warburg phenotype in tumors set the rationale for the use of ^18^F-fluorodeoxyglucose (^18^F-FDG) positron emission tomography (PET)/computed tomography (CT) in the diagnostic/prognostic clinical setting. However, unlike most solid tumors, primary PCa typically does not exhibit aerobic glycolysis and ^18^F-FDG-PET has shown limited utility in PCa detection. Glycolytic features have been nevertheless reported in high-risk PCa, where the acquisition of a Warburg phenotype marks a more aggressive disease. Pertega-Gomes *et al.*^[46]^ indeed observed a positive association between mRNA and protein levels of key glycolytic enzymes with worse clinicopathological features and biochemical recurrence (BCR). Accordingly, Choi and coworkers found high levels of lactate exporter monocarboxylate transporter 4 (MCT-4) protein in Gleason score (GS) 5 specimens, and again association with shorter time to develop BCR and CR^[11]^. Accordingly, high intraprostatic ^18^F-FDG uptake in high-risk PCa patients was reported as an indicator of shorter BCR free survival and shorter time to CR following radical prostatectomy^[47]^. These data support the potential use of preoperative ^18^F-FDG PET/CT as a non-invasive tool to distinguish patients in whom timely neo-adjuvant and adjuvant therapies should be explored.

The acquisition of a Warburg phenotype in mCRPC has been actively studied. Pertega-Gomes *et al*.^[46]^ reported increased mRNA and protein expression of key glycolytic enzymes, including lactate dehydrogenase A (LDHA) and MCT-4 in mCRPC human specimens. Similar results were observed in the metastatic/CR TRAMP murine model by Bok and coworkers using hyperpolarized (HP) ^13^C magnetic resonance spectroscopic imaging (MRSI) and multi-parametric ^1^H magnetic resonance imaging (MRI)^[48]^.

The first mechanistic explanation for a Warburg phenotype in the metastatic setting was provided by Diedrich and coworkers using a preclinical model of bone metastasis. According to the authors, bone marrow-enriched fat cells promote aerobic glycolysis in metastatic PCa cells via oxygen-independent HIF-1α activation and consequent induction of glycolytic enzymes^[34]^. While evidence for the same mechanism occurring in human mCRPC still need to be provided, these preliminary findings suggest a role for aerobic glycolysis in PCa progression/CR establishment and support the exploration of targeting LDHA and MCT transporters as new therapeutic opportunities for mCRPC, as discussed below.

Altogether, FA and glucose metabolism rewiring appears to be a recurrent feature associated with CR and resistance to ADT/AR signaling inhibitors, calling for the need of testing glycolysis and lipid metabolism modulators in combination with standard of care, as discussed below.

### Aerobic glycolysis and neuroendocrine PCa

The role of aerobic glycolysis in neuroendocrine PCa (NEPC) has been the object of recent investigations. This very aggressive and lethal PCa subtype presents unique features, including loss of AR signaling during neuroendocrine transdifferentiation after treatment with AR-targeting anti-androgens, resulting in CR. Using patient-derived xenografts (PDXs), tumor tissues and gene expression data, Choi and coworkers reported enhanced glycolytic features associated with increased lactic acid production/secretion in NEPC. MCT-4 expression silencing in NEPC NCI-H660 cells was reported to inhibit cell proliferation, suggesting that elevated glycolysis coupled to excessive MCT4-mediated lactic acid secretion may be clinically relevant to NEPC. Consequently, targeting MCT4 might be worth testing as a new therapeutic strategy for NEPC^[49]^. Discordant results were, however, reported by Zacharias *et al*.^[50]^ using *in vivo* HP MRSI, as well as lactate measurements *ex vivo*. The authors reported increased lactate production in AR-dependent CRPC PDX models compared to AR-negative neuroendocrine PDX models, highlighting the need for further experimental work to fully elucidate the role of aerobic glycolysis in NEPC and the clinical potential of therapeutic approaches that target it.

### Metabolic alterations beyond lipid and glucose metabolism and castration resistance

The advent of metabolomics technologies has allowed for the unbiased interrogation of cancer metabolism and the identification of metabolic pathways involved in CR, previously uncharacterized. In 2013, the group led by Chinnaiyan used high-throughput liquid and gas chromatography-based MS (LC/GC-MS) to profile the metabolome of 262 clinical samples (42 tissues, including 14 metastatic tissues, and 110 each of urine and plasma). Metabolic profiling was able to distinguish clinically localized PCa from mCRPC. Importantly, sarcosine, an N-methyl derivative of the amino acid glycine, emerged as a differential metabolite highly increased in mCRPC, and detectable non-invasively in urine. The authors went further to demonstrate AR binding to the promoter of the gene of glycine N-methyltransferase, the enzyme that coverts glycine into sarcosine, suggesting an interplay between AR and the sarcosine pathway^[51]^. Unfortunately, other studies did not confirm sarcosine as a biomarker for aggressive PCa, most likely due to the technical difficulties to accurately measure sarcosine, and the difference in the study population^[52]^. Thus, improvements in sarcosine detection and a careful design of prospective studies are still strongly needed before this biomarker can be used in precision medicine.

Following this first study, several groups have investigated the metabolic changes that support CR acquisition. In 2014, Kaushik and coworkers used a combination of targeted MS and metabolic phenotyping and identified nineteen metabolites that were altered in androgen-independent compared to androgen-dependent cell lines. These altered metabolites mapped to a highly interconnected network of biochemical pathways that describe UDP glucuronosyltransferase activity and were associated with time to treatment failure^[53]^. The year after, Shafi and al. reported an increase in glutaminolysis and reductive carboxylation in CRPC cell lines harboring AR-V7, suggesting their potential involvement in the resistance to endocrine therapies^[54]^. More recently, the group of Freedland combined metabolomics and lipidomics in patient serum and identified reduction in steroid synthesis, ketogenesis and hyperglycemia as prominent hallmarks of ADT treatment, suggesting the potential use of restricted ketogenic diets in improving ADT-linked comorbidities^[55]^. Altogether, these findings support a scenario where metabolic pathways other than FA and glucose metabolism may be altered in CRPC, suggesting the involvement of a more complex metabolic network in the acquisition of CR.

## Genetic drivers, metabolic reprogramming, and resistance to androgen therapies

Genomic analyses showed the molecular heterogeneity of mCRPC. The most common alterations are AR amplifications/mutations (63%), p53 loss (53%) PTEN loss (50%), MYC amplification (18%), PI3K mutations (< 15%), BRAC1 and BRAC2 mutations (14.6%)^[56]^. All these genetic alterations have been associated with resistance to endocrine therapies through different mechanisms, including metabolism reprogramming, highlighting the tight link between genetic drivers, metabolic alterations and CR. CR following PTEN loss has been associated with alterations in the metabolic flux of glycolysis, glutaminolysis, branched-chain amino acid catabolism and FA metabolism^[57]^. The latter has gained special attention since Chen *et al*.^[58]^ demonstrated that co-deletion of PTEN and promyelocytic leukemia protein (PML) activates mitogen-activated protein kinase, resulting in a SREBP-dependent lipogenic program that promotes mCRPC. Concordantly, HFD triggered SREBP-dependent lipogenesis and induced metastasis in the nonmetastatic PTEN knockout (KO) model and further enhanced metastasis in the PTEN/PML KO model, suggesting the lipogenic program as an underlying rheostat toward metastatic PCa progression. More recently, PTEN loss has been associated with CR via intratumoral androgen synthesis triggered by the KT-RUNX2-OCN-GPRC6A-CREB signaling axis, resulting in the overexpression of the *CYP11A1* and *CYP17A1* genes^[59]^.

The oncogene c-MYC (hereafter called MYC) is a well characterized master metabolic regulator. MYC controls a wide range of metabolic pathways, including glycolysis, glutaminolysis, FA metabolism (both synthesis and oxidation) and protein synthesis^[60,61]^. MYC is overexpressed early in the disease and amplified in about 20% of mCRPC, and its transcript levels are increased in AR-V7-high bone metastases^[56,62,63]^. MYC is an androgen-independent, AR-dependent targeted gene, whose protein overexpression induces androgen-independent PCa growth in preclinical models^[64]^. Recently, two studies showed MYC involvement in AR-V7 stabilization and in the regulation of alternative splicing, linking MYC with the acquisition of resistance to endocrine therapies^[65,66]^. In 2015, Mills and coworkers demonstrated MYC regulation of phosphoribosylaminoimidazole carboxylase (PAICS) and inosine monophosphate dehydrogenase 2 (IMPDH2), enzymes involved in *de novo* purine biosynthesis. High expression levels of the *PAICS* and *IMPDH2* genes were reported in mCRPC, while IMPDH2 inhibition with mycophenolic acid sensitized the response to AR signaling inhibitors, suggesting a potential role for PAICS and IMPDH2 in mediating the resistance to endocrine therapies^[67]^.

p53 is a tumor suppressor able to revert many of the metabolic effects exerted by MYC through inhibition of genes associated with glucose uptake, pentose phosphate shunt and nucleotide synthesis pathway^[68,69]^. p53 was reported to directly bind to the promoter region of SREBP-1, inducing gene silencing and *de novo* lipogenesis inhibition^[70]^. p53 inactivation was also associated with alterations in glucose, FA, oxidative phosphorylation, glutathione metabolism^[71]^ and inhibition of AR activity^[72]^. In 2018, Maughan and coworkers showed that p53 status in the primary PCa is predictive of inferior response to AR signaling inhibitors in mCRPC^[73]^, suggesting that p53 loss-associated metabolic alterations might be involved in the acquisition of resistance to AR signaling inhibitors.

PCa patients carrying BRCA 2 mutations become resistant to ADT faster than non-carriers^[74]^. Whether BRCA 2 mutation status affects the response to AR signaling inhibitors is still an object of investigation^[75]^. It has been reported that breast cancers harboring BRCA 1 mutations manifest a Warburg-like phenotype^[76]^, which might also characterize BRCA 2-mutated mCRPC. Future investigations are required to explore this aspect.

## Targeting metabolic vulnerabilities to overcome resistance to endocrine therapies

### Inhibitors of de novo fatty acid and cholesterol synthesis

As lipid metabolism rewiring, in the form of increased FA and cholesterol synthesis, uptake and oxidation, is a hallmark of mCRPC and resistance to endocrine therapies, efforts have focused on tackling enzymes and transporters involved in these processes.

FASN, the key enzyme in the synthesis of FA palmitate, is certainly the most studied and the best characterized therapeutic target. However, despite the positive results in the preclinical setting, off-target effects, poor solubility and pharmacokinetics, and untoward side effects, including important weight loss, have hampered the clinical translation^[77]^. This reality has recently changed with the development of TVB-2640, an orally available inhibitor of the FASN β-ketoacyl reductase domain. A phase I clinical trial has been recently completed proving the safety and efficacy of the drug in patients with advanced solid malignancies (NCT02223247). Combined with paclitaxel, TVB-2640 proved to be beneficial in heavily pretreated breast cancer patients, while the non-orally available analog TVB-3166 has shown promising results in preclinical models of mCRPC^[78-80]^. Phase II trials are now investigating TVB-2640 in several solid tumor types (NCT03032484, NCT03179904, NCT02980029 and NCT03808558) [Table 1].

**Table 1:** Promising metabolic drugs currently explored for the treatment of CRPC in combination with ADT, AR signaling inhibitors, or chemotherapy

Drug	Metabolic Target	Model	Anti-cancer effect	Drugs combination	Drug development timeline
Lipid metabolism					
TVB-3166	FASN	- mCRPC human cell lines - mCRPC xenografts	mCRPC cell lines - Reduction of AR/AR-V7 proteins - Reduction of proliferation/soft agar colony growth - Induction of apoptosis - Reduction of oncogenic signaling (i.e., b-catenin, c-MYC, *etc*.) CRPC xenografts - Reduction of xenograft tumor growth	TVB-3166+ paclitaxel - FASN inhibition-mediated increase of taxanes efficacy in CRPC	Preclinical - The orally available, TVB-2640 is currently tested in patients with various cancer types (Phase 2 trials: NCT03032484,NCT03179904,NCT02980029, NCT03808558)
IPI-9119	FASN	- mCRPC cell lines - mCRPC xenografts - Human mCRPC organoids	Androgen sensitive and mCRPC cell lines - Reduction of AR/AR-V7 proteins - Reduction of proliferation/soft agar colony growth - Cell cycle inhibition - Induction of apoptosis - Induction of endoplasmic reticulum (ER) stress - Reduction of protein synthesis mCRPC xenografts - Reduction of xenograft tumor growth Human mCRPC organoids - Reduction of mCRPC organoid growth	IPI-9119+ enzalutamide mCRPC cell lines - FASN inhibition-mediated increase of enzalutamide efficacy in mCRPC cells	Preclinical
Atorvastatin	HMGCR	Humans	NA. Recruiting stage. Primary objective - This randomized double-blind placebo-controlled trial is designed to explore whether the intervention with atorvastatin delays PCa progression (i.e., development of CR compared to placebo during ADT for metastatic or recurrent PCa)	Atorvastatin+ADT	Phase 3 trial (NCT04026230)
Mevastatin Simvastatin	HMGCR	- mCRPC cell lines - mCRPC xenografts	mCRPC cell lines - Reduction of AR/AR-Vs proteins via inhibition of mTOR pathway - Reduction of proliferation - Induction of apoptosis - Reduction of p-mTOR, p-Akt, p-S6RP mCRPC xenografts - Reduction of xenograft tumor growth - Reduction of proliferative rate - Increase of apoptotic rate	Simvastatin+enzalutamide mCRPC xenografts - Simvastatin-mediated increase of enzalutamide efficacy in mCRPC - Higher reduction of proliferative rate in the combo - Higher increase of apoptotic rate in the combo	Preclinical
BMS-303141	ACLY	- mCRPC cell lines - mCRPC xenografts	mCRPC cell lines - Reduction of proliferation - Reduction of AR protein - Induction of apoptosis - Activation of AMPK - Induction of ER stress	BMS-303141+enzalutamide - BMS-303141-mediated increase of enzalutamide efficacy in mCRPC via AMPK activation - Dramatic suppression of AR and AR target gene expression - Higher reduction of proliferative rate in the combo - Higher increase of apoptotic rate in the combo - Stronger induction of ER stress in the combo	Preclinical
Etomoxir	CPT-1	- mCRPC cell lines	- Reduction of proliferation/soft agar colony growth	Etomoxir+Enzalutamide - Etomoxir-mediated increase of Enzalutamide efficacy in mCRPC cells - Higher reduction of proliferative rate/ soft agar growth in the combo	Preclinical - The clinical translation of Etomoxir has been terminated due to its toxic side effects
Ranolazine	3-KAT*	- mCRPC cell lines - mCRPC xenografts	mCRPC cell lines - Reduction of proliferation/soft agar colony growth	Ranolazine+enzalutamide mCRPC cell lines - Ranolazine-mediated increase of enzalutamide efficacy in mCRPC cells - Higher reduction of proliferative rate/ soft agar growth in the combo mCRPC xenografts - Higher reduction of proliferative rate in the combo	Preclinical #
Perhexilline	CPT-1	- mCRPC cell lines	- Reduction of proliferation/soft agar colony growth	Perhexilline+enzalutamide - Perhexilline-mediated increase of enzalutamide efficacy - Higher reduction of proliferative rate/ soft agar growth in the combo	Preclinical #
Glucose metabolism					
AR-C155858	MCT-1	- *Ex-vivo* tissue slices of human PCas	- Reduction of proliferative rate - Increase of apoptotic rate		Preclinical - AstraZeneca MCT-1 inhibitor (AZD3965) is currently tested in patients with advanced solid tumors (Phase I trial: NCT01791595)
FX11	LDHA	- ATM-deficient mCRPC cells - ATM-deficient mCRPC xenografts	ATM-deficient mCRPC cell lines - Reduction of viability rate - Increase of ROS levels ATM-deficient mCRPC xenograft - Reduction of tumor growth in FX11-treated group		Preclinical
Gossypol (AT-101)	LDHA	- Humans	Study No. 1: Test of AT-101 and ADT in patients with newly diagnosed metastatic PCa Study No. 2: Comparison of AT-101 with docetaxel and prednisone *vs.* docetaxel and prednisone alone in men with chemotherapy-naïve metastatic hormone refractory PCa (HRPC) Study No. 3: Test of AT-101 in men with HRPC Study No. 4: Test the safety and efficacy of AT-101 in combination with docetaxel and prednisone in men with HRPC	Primary objectives: Study No. 1: Determine the % of patients with newly diagnosed metastatic PCa with undetectable PSA (< 0.2 ng/mL) at 7 months following treatment with AT-101 and ADT Study No. 2: Compare the two treatment arms with respect to overall survival (time frame: 33 months) Study No. 3. Determine the number of participants treated with AT-101 showing adverse events Study No. 4: Determine the safety of AT-101 in combination with docetaxel and prednisone (time frame: 12 months)	Study No. 1: Phase 2 trial: NCT00666666 Study No. 2: Phase 2 trial: NCT00571675 Study No. 3: Phases 1/2 trial: NCT00286806 Study No. 4: Phases 1/2 trial: NCT00286793

*3-KAT: 3-ketoacylthiolase; ^#^These drugs are already approved in Europe, US, and Australia for the treatment of heart diseases in patients

In the context of mCRPC, our group characterized a new small-molecule FASN inhibitor (IPI-9119) and demonstrated that selective FASN inhibition antagonizes the growth of mCRPC in *in vitro* models through metabolic reprogramming and results in reduced protein expression and transcriptional activity of both AR-FL and AR-V7. Interestingly, FASN inhibition in mCRPC cells harboring AR-V7 downregulated especially the variant, suggesting a distinctive interplay between AR-V7 and *de novo* lipogenesis. Mechanistically, FASN inhibition triggered the activation of the endoplasmic reticulum stress response, resulting in reduced protein synthesis. Our analysis also demonstrated consistent inhibition of MYC and the MYC-mediated transcriptional program, following FASN suppression. As mentioned above, MYC promotes CR and AR-V7 stabilization and competes with AR for binding to AR-regulated genes. Thus, the opportunity to repress AR/AR-V7 and MYC signaling simultaneously is particularly attractive in the mCRPC setting. *In vivo*, IPI-9119 reduced the growth of AR-V7-driven mCRPC xenografts and human mCRPC-derived organoids. IPI-9119 and enzalutamide combination induced higher mCRPC cell growth inhibition than single treatment. Thus, FASN targeting represents a non-canonical approach to inhibit AR/AR-V7 signaling and to improve the activity of AR signaling inhibitors. In human mCRPC, FASN was co-expressed with AR-FL in 87% of metastases and with AR-V7 in 39% of bone metastases. In patients treated with enzalutamide and/or abiraterone, FASN/AR-V7 double-positive metastases were found in 77% of cases, providing a compelling rationale for the use of FASN inhibitors in mCRPCs^[42]^. We envisage that IPI-9119 could be combined with AR signaling inhibitors in AR-V7-negative CRPC to delay/overcome resistance. Alternatively, FASN inhibition could be undertaken in tumors (AR-V7-positive) once resistance has already emerged and further therapeutic options are scarce. Carefully designed clinical trials are required to establish the therapeutic timing, combinatorial regimens and the suitable population to treat.

Inhibition of HMGCR, a key enzyme in the mevalonate pathway for sterol biosynthesis, is currently being tested in a phase 3 clinical trial. This double-blind placebo-controlled trial, currently recruiting patients, is designed to test whether atorvastatin (an HMGCR inhibitor) delays the development of CR during ADT in metastatic or recurrent PCa (NCT04026230). In the preclinical setting, HMGCR inhibition with simvastatin enhanced the efficacy of enzalutamide-based therapy and decreased AR/AR-V7 protein levels via inhibition of the mTOR pathway^[43]^.

The clinical translation of statins in oncology has been easier than for other compounds since they are relatively safe and already used for the treatment of hypercholesterolemia in patients. A recent meta-analysis evaluated the effects of statin use on treatment outcomes (i.e., overall survival and cancer-specific survival) among patients with advanced PCa treated with ADT or AR signaling inhibitors. Statin use was associated with lower risk of all-cause mortality (HR = 0.73; 95%CI: 0.64-0.83; *P* < 0.00001) and cancer-specific mortality (HR = 0.64; 95%CI: 0.53-0.77; *P* < 0.00001) in advanced PCa patients treated with ADT, whereas inconsistent results were obtained with AR signaling inhibitors. Thus, future studies are still required to establish the efficacy of statins in combination with AR signaling inhibitors in mCRPC patients^[81]^.

Targeting ACLY, the enzyme that converts citrate to acetyl-CoA for FA synthesis, has shown promising results in the preclinical setting^[40]^. In particular, the group led by Wellen demonstrated that ACLY inhibition sensitizes mCRPC cells to enzalutamide by impinging on an ACLY- 5’ AMP-activated protein kinase (AMPK)-AR feedback mechanism. The combination of ACLY inhibitor BMS-303141 and enzalutamide promoted energetic stress and AMPK activation, resulting in further suppression of AR levels and target gene expression, inhibition of proliferation, and apoptosis. These data suggest that the ACLY-AMPK-AR network could be exploited to sensitize CRPC cells to AR antagonism^[82]^. Unfortunately, no ACLY inhibitors have reached the clinical level so far [Table 1].

### Inhibitors of fatty acid elongation and oxidation

Promising results have also been obtained by gene silencing of ELOVL 5 and 7^[83,84]^, but again, none of the available ELOVL inhibitors has been suitable for clinical studies.

As mentioned above, targeting FAO as a therapeutic approach in mCRPC has recently gained interest. Iglesias-Gato and coworkers performed a proteomics analysis and identified a subgroup of bone metastases characterized by elevated expression of many canonical AR targets and FAO enzymes, highlighting the need for adequate patient stratification when metabolic therapies are considered as therapeutic approach^[85]^. Combinations of FAO inhibitors (etomoxir, ranolazine and perhexiline) and enzalutamide were tested in mCRPC cell and xenograft models. The effects of the combinations were very strong with the irreversible inhibitor etomoxir and robust, albeit less potent, with ranolazine and perhexiline^[86]^. While the clinical translation of etomoxir has been terminated due to the toxic side effects (mostly hepatotoxicity), ranolazine and perhexiline are already approved in Europe, US, and Australia for the treatment of heart diseases, thus opening a potential safe avenue for the combinations of FAO inhibitors and AR signaling inhibitors [Table 1].

### Inhibitors of aerobic glycolysis and lactate shuttle

While the majority of efforts have been focused on lipid metabolism, the therapeutic potential of inhibiting the Warburg phenotype of mCRPC (see above) is currently in the limelight. Genetic silencing or pharmacological inhibition of LDHA or lactate transporters MCT-1/MCT-4 showed promising results in mCRPC cell lines and murine models^[46,87,88]^. LDHA knockdown or inhibition with the compound FX11 was particularly effective in the treatment of ATM-deficient CRPC cells, suggesting that targeting glycolysis may be beneficial for patients with PARP inhibitor-resistant mCRPC^[89]^. The NADH-competitive LDHA inhibitor gossypol (AT-101) is currently tested in patients with newly diagnosed metastatic PCa and CRPC (NCT00666666, NCT00571675, NCT00286806 and NCT00286793). The drug is relatively safe and effective in reducing tumor markers^[90]^; however, it lacks selectivity. The drug was indeed reported to antagonize the anti-apoptotic BCL2 family of proteins by acting as a BH3 domain mimetic, and thus, the antitumor properties observed with AT-101 are likely due to suppression of both targets. Regarding MCT inhibitors, AstraZeneca has recently developed a specific MCT-1 inhibitor (AZD3965) that is currently in a phase 1 clinical trial (NCT01791595), while MCT-4 inhibitors are still in the discovery phase [Table 1]. The future development of selective LDHA and MCT-4 inhibitors will be crucial to understand the clinical value of targeting aerobic glycolysis in mCRPC.

## TME-PCa cell metabolic crosstalk

Recent insights into the critical role of TME in disease progression underscored the need to investigate how metabolic reprogramming in cancer-associated fibroblasts (CAFs), adipocytes and infiltrating immune cells contributes to disease progression and resistance to ADT/AR signaling inhibitors. Several studies have been conducted to address this aspect. Valencia and coworkers documented a reduction in p62 levels in PCa stroma. The authors proposed that tumor epithelial cells favor the downregulation of p62 in CAFs leading to reduced mTORC1 activity and c-Myc expression, impaired metabolic detoxification, and release of IL-6 with subsequent promotion of epithelial tumor growth and invasion^[91]^. Mishra and coworkers identified epigenetic changes in CAFs that promote a cascade of stromal-epithelial metabolic interactions and facilitate lethal PCa growth and ADT resistance^[92]^. Two independent studies also highlighted the existence of a metabolic crosstalk via the lactate shuttle between PCa cells and CAFs to sustain cancer aggressiveness. Ippolito *et al*.^[93]^ reported increased release and uptake of lactate from CAFs and PCa cells, respectively, resulting in lactate oxidation and alteration of NAD+/NADH ratio in PCa cells, followed by SIRT1-dependent PGC-1α activation, increased mitochondrial activity, superoxide generation and growth advantage. Accordingly, Pertega-Gomes observed the expression of MCT-1 and MCT-4 lactate transporters in the epithelium and stromal compartment of human PCa tissues, respectively. High stromal MCT-4 and high epithelial MCT-1 were associated with poor outcome, highlighting the clinical relevance of this lactate shuttle in the clinical setting^[94]^.

While still in its infancy, a better understanding of the metabolic crosstalk (metabolic symbiosis and antagonism) between TME and PCa cells might thus be critical in identifying novel mechanisms of CR and vulnerabilities that can be exploited therapeutically.

The integration of patient-derived explants, PDX models and stroma/epithelium co-culture models using human PCa organoids will be crucial in elucidating the metabolic interactions between PCa cells and TME in systems that more closely recapitulate PCa heterogeneity and complexity.

## Potential contribution of obesity and diet to endocrine therapy resistance

Several epidemiological studies have documented the association between obesity and lethal PCa^[95]^. A recent paper demonstrated that this association occurs regardless of race^[14]^. Whether unhealthy diets, especially fat-rich ones promote PCa progression is still a matter of debate, despite that is has been consistently observed in preclinical models^[12,40]^. Inconsistent results in human studies may be due to population selection and/or difference in dietary interventions between studies.

Both obesity and sustained consumption of fat-rich diet alter the nutrient gradient in the TME, which may favor cancer cells/TME metabolic symbiosis, inflammation, cancer progression and chemoresistance^[96]^.

As previously outlined, Chen *et al*.^[58]^ demonstrated that a saturated FA-enriched diet was sufficient to promote mCRPC in a nonmetastatic PTEN-null model via an aberrant lipogenic program orchestrated by the transcription factor SREBP. Consistently, our group demonstrated that increased intake of saturated FAs enhances a c-MYC-mediated oncogenic transcriptional program, which is associated with lethality in patients^[97]^. In 2004, Ngo and coworkers showed that reduced dietary fat intake delays the progression to CRPC in xenograft models and prolongs survival, suggesting low-fat diet as a promising adjuvant intervention during ADT^[98]^. The impact of a carbohydrate rich-diet on PCa progression has also started to be recently investigated^[99]^. Freedland and coworkers reported a randomized controlled trial (CAPS1) where the combination of a low carbohydrate diet (LCD) (≤ 20 g carbohydrate/day) plus walking (≥ 30 min for ≥ 5 days/week) was an effective strategy for blocking ADT adverse metabolic effects^[100]^. In a second study (CAPS2), the same authors reported exploratory findings of longer PSA doubling time under LCD compared to control in patients with BCR, calling for larger studies testing LCD on disease progression^[101]^. Clinical trials are currently ongoing to evaluate the efficacy of fat- and sugar-restricted diet on PCa progression and mCRPC treatment. Results from these studies will be valuable to determine whether nutrition intervention has a beneficial effect on PCa progression and whether it should be implemented in the management of PCa patients prior to or along with ADT/AR signaling inhibitors.

Furthermore, when targeting lipogenic enzymes is considered a potential therapeutic approach, nutritional parameters and obesity status should be recorded to monitor for potential lipid uptake compensation due to increased systemic and TME nutrient availability^[96]^.

## Metabolomics and lipidomics in PCa precision medicine

**Figure 3:**
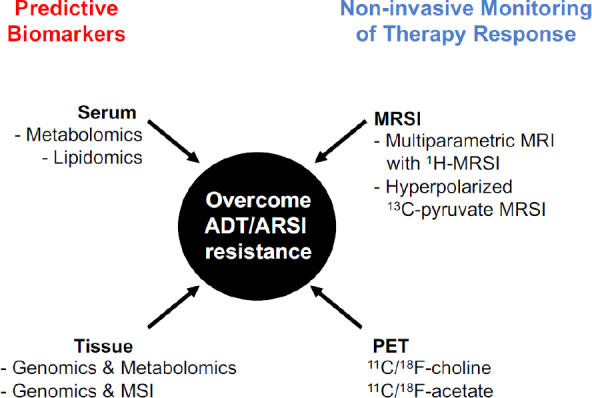
Metabolomics and metabolic imaging applications in PCa. Schematic overview of metabolomics and metabolic imaging applications in biomarker discovery and in therapy response assessment. ADT: androgen deprivation therapy; ARSI: androgen receptor signaling inhibitors; MSI: mass spectrometry imaging; MRI: magnetic resonance imaging; MRSI: magnetic resonance spectroscopy imaging; PET: positron emission tomography

The success of targeted therapies lies on the selection of patients most likely to respond and the timely evaluation of therapy response in a non-invasive manner. The last decade has seen enormous progress in the application of metabolomics and metabolic imaging to identify biomarkers of disease aggressiveness and response to ADT/AR signaling inhibitors [Figure 3].

Two recent systematic reviews have summarized the results of current efforts in using metabolomics to identify predictive, diagnostic, prognostic and treatment response biomarkers. Kelly *et al*.^[102]^ described thirty-three human case-control studies. All but one demonstrated the ability of metabolite profiling in correctly identifying cancer *vs*. benign, tumor by aggressiveness, cases that recurred, and those that responded well to therapy. Biomarker discriminatory ability was quantified for some metabolites, reporting areas under the curve that would potentially outperform the current gold standards. Following the work of Kelly *et al*.^[102]^, Kandra and coworkers provided an updated analysis that included fifty-nine studies. Some discriminative metabolites of PCa disease were reported across various technical approaches, cohorts and sample types, suggesting their potential clinical relevance. Glycine, in particular, was repeatedly increased in both biological fluids and tumor tissues of PCa patients, requiring further investigation as potential diagnostic biomarker. So far, only few studies focused on metabolic biomarkers of disease progression and treatment response to ADT have been conducted. Lower levels of citrate and spermine in PCa tissues were found predictive of worse prognosis^[103]^, while deoxycholic acid, glycochenodeoxycholate, docosapentaenoic acid, tryptophan, arachidonic acid, the nucleotide deoxycytidine triphosphate and pyridinoline were identified as potential biomarkers for predicting response to ADT^[104]^. Caution, however, must be taken in interpreting these studies. Lack of replication and validation remains the biggest limitation in the field of metabolomics, an issue that has prevented the use of metabolic biomarkers in the clinical routine so far. Moreover, pre-analytical and technical obstacles, statistical artifacts and confounding factors are still unresolved issues. Carefully designed future studies with appropriate sample size, controls in place at the pre-analytical, analytical, and clinical stage, and finding validation using an appropriate set of independent samples are absolutely required prior the integration of metabolomics in the practice of precision medicine.

Despite the crucial role of lipid metabolism in PCa progression and resistance to endocrine therapies, lipidomics studies have only recently been reported in the literature, most likely due to the methodological challenge of analyzing simultaneously diverse lipid classes and molecular species. However, these studies are now becoming increasingly popular in the field of biomarker discovery. Lin *et al*.^[105]^ performed LC/MS- based lipidomic profiling on plasma samples from a discovery cohort of CRPC patients and validated the results in an independent cohort. Unsupervised analysis of lipidomic profiles classified the discovery cohort into two subgroups with significant survival differences (HR = 2.31; 95%CI: 1.44-3.68; *P* = 0.0005). Forty-six lipids, predominantly sphingolipids, were associated with poor prognosis. The authors were able to derive and validate a prognostic three-lipid signature (ceramide d18:1/24:1, sphingomyelin d18:2/16:0 and phosphatidylcholine 16:0/16:0) as independent prognostic factor^[105]^. Moreover, the group of Butler used lipidomics to profile PCa cell lines, xenografts and patient-derived explants under treatment with androgen and AR signaling inhibitors. The authors identified changes in lipid elongation for multiple phospholipid classes in response to androgen treatment, which was reversed by enzalutamide, suggesting the utility of lipidomics to predict response to endocrine therapies^[83]^.

While the high resolution, sensitivity and specificity of LC/MS-based metabolomics and lipidomics have favored their use in biomarker discovery, these technologies, which analyze metabolite extracts, fail to provide spatial information, preventing the possibility to interrogate cancer biomarkers in relation to tissue pathology and compartment distribution. The development of MSI has overcome this limitation, allowing the overlap of metabolic and pathology information on the same tissue section. MSI thus represents an important step forward for the evaluation of metabolic reprogramming occurring in the TME. Matrix-assisted laser desorption ionization (MALDI)-MSI, where the sample is mixed with a UV-absorbing crystalline matrix material and ionized by the laser beam, is the most commonly used MSI method. Our group has recently applied MALDI-MSI to investigate prognostic biomarkers in prostatectomy samples with GS of six to nine. A gradient of changes in the intensity of various lipids was observed, which correlated with increasing GS. Moreover, a total of thirty-one lipids, including several phosphatidylcholines, phosphatidic acids, phosphatidylserines, phosphatidylinositols, and cardiolipins were able to discriminate GS (4+3) from GS (3+4) cases, suggesting their potential clinical relevance in predicting more aggressive/lethal PCa in intermediate risk cases based on pathology^[106]^. Given the promising results with MALDI-MSI, this technology holds great promise in the identification of predictive biomarkers to ADT response by interrogating both cancer epithelium and stroma compartment. Advances are also being made in “omics” integration and, more recently, in spatial metabolomics and transcriptomics integration^[107-109]^. While still challenging, metabolomics/transcriptomics integration could offer data-driven system approaches to refine our understanding of mechanisms of CR.

In the context of non-invasive imaging biomarkers, Kurhanewicz *et al*.^[110]^ have been at the front line in the application of MRSI in PCa. The advantage of this technology with respect to MSI is that it can be applied both *ex vivo* and *in vivo* in tandem with MRI. Compared to PET which measures the retention of a single radiolabeled metabolite, ^1^H-MRSI (the MRSI method most commonly used in the clinical setting) is a radiation-free technique that allows *in vivo* measurement of a complete spectrum of measurable metabolites to distinguish tumor areas, inform on cancer aggressiveness, and monitor therapy response. The detection of high choline (Cho) and creatine (Cr) levels and reduced citrated (Cit) and polyamines measured by ^1^H-MRSI has been used for years to support PCa diagnosis using multiparametric MRI, where Cho+Cr/Cit magnitude is a parameter of disease aggressiveness^[111]^.

Dr. Kurhanewicz has also pioneered the development and clinical translation of an improved MRSI technology utilizing hyperpolarized (HP) ^13^C-labeled metabolic substrates for the rapid and non-invasive monitoring of dynamic pathway-specific metabolic processes. Hyperpolarization, achieved through the dynamic nuclear polarization technique, provides unprecedented gain in sensitivity (10,000- to 100,000-fold increase) for imaging ^13^C-labeled biomolecules that are endogenous, nontoxic and nonradioactive. Metabolically active HP ^13^C-labeled compounds can be delivered to living systems where the substrate is metabolized, and the products can be imaged in real time. While the metabolism of primary PCa is inadequately evaluated using ^18^F-FDG PET (which measures glucose uptake and phosphorylation)^[112]^, HP ^13^C-MRSI detects downstream metabolism, specifically the conversion of HP [1-^13^C] pyruvate to [1-^13^C] lactate catalyzed by LDHA. The authors reported the first-in-human phase 1 clinical study of HP ^13^C-pyruvate MRSI in patients with PCa on active surveillance and confirmed the feasibility of capturing regions of accelerated HP pyruvate-to-lactate flux in high-grade *vs*. low-grade cancer *vs*. benign tissue^[113]^. Most recently, the same group used HP ^13^C-pyruvate MRI in patients with mCRPC^[114]^ and described the feasibility of assessing metabolic response to ADT. Strikingly, these authors showed the ability of HP ^13^C MRI to detect early metabolic responses before such a response could be ascertained using standard radiographic criteria^[115]^. These findings highlight the great potential of using HP-MRSI and MRI to non-invasively monitor early response to endocrine therapies in PCa patients. A prospective imaging study evaluating the utility of baseline metabolic MRI with HP ^13^C-pyruvate as a predictive response biomarker to AR signaling inhibitors in CRPC patients is currently ongoing (NCT02911467).

While limited by the use of radiotracers and the need of a cyclotron for radiotracer synthesis, ^11^C-choline- and ^11^C-acetate-based PET has also shown promising results in detecting recurrent disease and in monitoring response to ADT^[116,117]^ and it can very be useful in future studies, particularly in evaluating response to anti-lipogenic drugs and ADT combination.

## Conclusion

The identification of a mutual interplay between cell metabolism reprogramming and AR signaling has opened new opportunities in mCRPC treatment and in monitoring the response to endocrine therapies. In this new scenario, we foresee the use of metabolomics approaches for patient stratification, the use of metabolic modulators in combination with standard of care to overcome drug resistance, and the use of metabolic imaging to predict and to monitor, non-invasively, therapy response. Successful therapeutic strategies also need to take into account TME heterogeneity and the dynamic changes in nutrient gradients, particularly in response to therapy. The use of accurate models that more faithfully resemble tumor heterogeneity such as patient-derived explants, PDX, and stromal co-culture with human organoids will be critical in defining the benefits of targeting metabolic pathways along with ADT or AR signaling inhibitors.

Well-designed clinical trials with sufficient statistical power and adequate validation are also in urgent need to elucidate whether obesity and the consumption of unbalanced diets may alter the response to ADT or AR signaling inhibitors and contribute to the acquisition of CR.

In a time where metabolic drugs have finally reached the clinical setting and metabolomics and imaging technologies are constantly improving, we are seeing the exciting opportunity to evaluate the modulation of cancer metabolism as a strategy to improve the management of mCRPC patients.

## Declarations

### Copyright

© The Author(s) 2021.

## References

[1] Siegel RL, Miller KD, Jemal A (2020). Cancer statistics, 2020.. CA Cancer J Clin.

[2] Schrecengost R, Knudsen KE (2013). Molecular pathogenesis and progression of prostate cancer.. Semin Oncol.

[3] Teo MY, Rathkopf DE, Kantoff P (2019). Treatment of advanced prostate cancer.. Annu Rev Med.

[4] Einstein DJ, Arai S, Balk SP (2019). Targeting the androgen receptor and overcoming resistance in prostate cancer.. Curr Opin Oncol.

[5] Graf RP, Hullings M, Barnett ES, Carbone E, Dittamore R, Scher HI (2020). Clinical utility of the nuclear-localized AR-V7 biomarker in circulating tumor cells in improving physician treatment choice in castration-resistant prostate cancer.. Eur Urol.

[6] Salami J, Alabi S, Willard RR (2018). Androgen receptor degradation by the proteolysis-targeting chimera ARCC-4 outperforms enzalutamide in cellular models of prostate cancer drug resistance.. Commun Biol.

[7] Han X, Wang C, Qin C (2019). Discovery of ARD-69 as a highly potent proteolysis targeting chimera (PROTAC) degrader of androgen receptor (AR) for the treatment of prostate cancer.. J Med Chem.

[8] Neklesa T, Snyder LB, Willard RR (2019). ARV-110: an oral androgen receptor PROTAC degrader for prostate cancer.. J Clin Oncol.

[9] Kregel S, Wang C, Han X (2020). Androgen receptor degraders overcome common resistance mechanisms developed during prostate cancer treatment.. Neoplasia.

[10] Lin D, Ettinger SL, Qu S (2017). Metabolic heterogeneity signature of primary treatment-naïve prostate cancer.. Oncotarget.

[11] Choi SY, Xue H, Wu R (2016). The MCT4 Gene: a novel, potential target for therapy of advanced prostate cancer.. Clin Cancer Res.

[12] Wilson KM, Mucci LA, Dehm SM, Tindall DJ (2019). Diet and lifestyle in prostate cancer.. Prostate cancer..

[13] Lin PH, Aronson W, Freedland SJ (2019). An update of research evidence on nutrition and prostate cancer.. Urol Oncol.

[14] Vidal AC, Oyekunle T, Howard LE (2020). Obesity, race, and long-term prostate cancer outcomes.. Cancer.

[15] Keto CJ, Aronson WJ, Terris MK (2012). Obesity is associated with castration-resistant disease and metastasis in men treated with androgen deprivation therapy after radical prostatectomy: results from the SEARCH database.. BJU Int.

[16] Barfeld SJ, Itkonen HM, Urbanucci A, Mills IG (2014). Androgen-regulated metabolism and biosynthesis in prostate cancer.. Endocr Relat Cancer.

[17] Costello LC, Franklin RB (2016). A comprehensive review of the role of zinc in normal prostate function and metabolism; and its implications in prostate cancer.. Arch Biochem Biophys.

[18] Massie CE, Lynch A, Ramos-Montoya A (2011). The androgen receptor fuels prostate cancer by regulating central metabolism and biosynthesis.. EMBO J.

[19] Heemers HV, Verhoeven G, Swinnen JV (2006). Androgen activation of the sterol regulatory element-binding protein pathway: Current insights.. Mol Endocrinol.

[20] Chan SC, Selth LA, Li Y (2015). Targeting chromatin binding regulation of constitutively active AR variants to overcome prostate cancer resistance to endocrine-based therapies.. Nucleic Acids Res.

[21] Sharma NL, Massie CE, Ramos-Montoya A (2013). The androgen receptor induces a distinct transcriptional program in castration-resistant prostate cancer in man.. Cancer Cell.

[22] Mah C, Nassar ZD, Swinnen JV, Butler L (2020). Lipogenic effects of androgen signaling in normal and malignant prostate.. Asian J Urol.

[23] Swinnen JV, Van Veldhoven PP, Esquenet M, Heyns W, Verhoeven G (1996). Androgens markedly stimulate the accumulation of neutral lipids in the human prostatic adenocarcinoma cell line LNCaP.. Endocrinology.

[24] Swinnen JV, Brusselmans K, Verhoeven G (2006). Increased lipogenesis in cancer cells: new players, novel targets.. Curr Opin Clin Nutr Metab Care.

[25] Hosios AM, Vander Heiden MG (2018). The redox requirements of proliferating mammalian cells.. J Biol Chem.

[26] Wu X, Dong Z, Wang CJ (2016). FASN regulates cellular response to genotoxic treatments by increasing PARP-1 expression and DNA repair activity via NF-κB and SP1.. Proc Natl Acad Sci U S A.

[27] Ackerman D, Simon MC (2014). Hypoxia, lipids, and cancer: surviving the harsh tumor microenvironment.. Trends Cell Biol.

[28] Swinnen JV, Ulrix W, Heyns W, Verhoeven G (1997). Coordinate regulation of lipogenic gene expression by androgens: evidence for a cascade mechanism involving sterol regulatory element binding proteins.. Proc Natl Acad Sci U S A.

[29] Swinnen JV, Heemers H, van de Sande T (2004). Androgens, lipogenesis and prostate cancer.. J Steroid Biochem Mol Biol.

[30] Watt MJ, Clark AK, Selth LA (2019). Suppressing fatty acid uptake has therapeutic effects in preclinical models of prostate cancer.. Sci Transl Med.

[31] Tousignant KD, Rockstroh A, Taherian Fard A (2019). Lipid uptake is an androgen-enhanced lipid supply pathway associated with prostate cancer disease progression and bone metastasis.. Mol Cancer Res.

[32] Schlaepfer IR, Rider L, Rodrigues LU (2014). Lipid catabolism via CPT1 as a therapeutic target for prostate cancer.. Mol Cancer Ther.

[33] Itkonen HM, Brown M, Urbanucci A (2017). Lipid degradation promotes prostate cancer cell survival.. Oncotarget.

[34] Diedrich JD, Rajagurubandara E, Herroon MK (2016). Bone marrow adipocytes promote the Warburg phenotype in metastatic prostate tumors via HIF-1alpha activation.. Oncotarget.

[35] Stoykova GE, Schlaepfer IR (2019). Lipid metabolism and endocrine resistance in prostate cancer, and new opportunities for therapy.. Int J Mol Sci.

[36] Ettinger SL, Sobel R, Whitmore TG (2004). Dysregulation of sterol response element-binding proteins and downstream effectors in prostate cancer during progression to androgen independence.. Cancer Res.

[37] Sharp A, Coleman I, Yuan W (2019). Androgen receptor splice variant-7 expression emerges with castration resistance in prostate cancer.. J Clin Invest.

[38] Han W, Gao S, Barrett D (2018). Reactivation of androgen receptor-regulated lipid biosynthesis drives the progression of castration-resistant prostate cancer.. Oncogene.

[39] Joshi M, Stoykova GE, Salzmann-Sullivan M (2019). CPT1A supports castration-resistant prostate cancer in androgen-deprived conditions.. Cells.

[40] Zadra G, Loda M (2018). Metabolic vulnerabilities of prostate cancer: diagnostic and therapeutic opportunities.. Cold Spring Harb Perspect Med.

[41] Li X, Chen YT, Hu P, Huang WC (2014). Fatostatin displays high antitumor activity in prostate cancer by blocking SREBP-regulated metabolic pathways and androgen receptor signaling.. Mol Cancer Ther.

[42] Zadra G, Ribeiro CF, Chetta P (2019). Inhibition of de novo lipogenesis targets androgen receptor signaling in castration-resistant prostate cancer.. Proc Natl Acad Sci U S A.

[43] Kong Y, Cheng L, Mao F (2018). Inhibition of cholesterol biosynthesis overcomes enzalutamide resistance in castration-resistant prostate cancer (CRPC).. J Biol Chem.

[44] Warburg O (1956). On the origin of cancer cells.. Science.

[45] San-Millan I, Brooks GA (2017). Reexamining cancer metabolism: lactate production for carcinogenesis could be the purpose and explanation of the Warburg Effect.. Carcinogenesis.

[46] Pertega-Gomes N, Felisbino S, Massie CE (2015). A glycolytic phenotype is associated with prostate cancer progression and aggressiveness: a role for monocarboxylate transporters as metabolic targets for therapy.. J Pathol.

[47] Lavallee E, Bergeron M, Buteau FA (2019). Increased prostate cancer glucose metabolism detected by (18)F-fluorodeoxyglucose positron emission tomography/computed tomography in localised Gleason 8-10 prostate cancers identifies very high-risk patients for early recurrence and resistance to castration.. Eur Urol Focus.

[48] Bok R, Lee J, Sriram R (2019). The role of lactate metabolism in prostate cancer progression and metastases revealed by dual-agent hyperpolarized (13)C MRSI.. Cancers (Basel).

[49] Choi SYC, Ettinger SL, Lin D (2018). Targeting MCT4 to reduce lactic acid secretion and glycolysis for treatment of neuroendocrine prostate cancer.. Cancer Med.

[50] Zacharias N, Lee J, Ramachandran S (2019). Androgen receptor signaling in castration-resistant prostate cancer alters hyperpolarized pyruvate to lactate conversion and lactate levels in vivo.. Mol Imaging Biol.

[51] Sreekumar A, Poisson LM, Rajendiran TM (2009). Metabolomic profiles delineate potential role for sarcosine in prostate cancer progression.. Nature.

[52] Jentzmik F, Stephan C, Lein M (2011). Sarcosine in prostate cancer tissue is not a differential metabolite for prostate cancer aggressiveness and biochemical progression.. J Urol.

[53] Kaushik AK, Vareed SK, Basu S (2014). Metabolomic profiling identifies biochemical pathways associated with castration-resistant prostate cancer.. J Proteome Res.

[54] Shafi AA, Putluri V, Arnold JM (2015). Differential regulation of metabolic pathways by androgen receptor (AR) and its constitutively active splice variant, AR-V7, in prostate cancer cells.. Oncotarget.

[55] Chi JT, Lin PH, Tolstikov V (2020). Metabolomic effects of androgen deprivation therapy treatment for prostate cancer.. Cancer Med.

[56] Robinson D, Van Allen EM, Wu YM (2015). Integrative clinical genomics of advanced prostate cancer.. Cell.

[57] Zhou X, Yang X, Sun X (2019). Effect of PTEN loss on metabolic reprogramming in prostate cancer cells.. Oncol Lett.

[58] Chen M, Zhang J, Sampieri K (2018). An aberrant SREBP-dependent lipogenic program promotes metastatic prostate cancer.. Nat Genet.

[59] Yang Y, Bai Y, He Y (2018). PTEN loss promotes intratumoral androgen synthesis and tumor microenvironment remodeling via aberrant activation of RUNX2 in castration-resistant prostate cancer.. Clin Cancer Res.

[60] Dang CV (2013). MYC, metabolism, cell growth, and tumorigenesis.. Cold Spring Harb Perspect Med.

[61] Dejure FR, Eilers M (2017). MYC and tumor metabolism: chicken and egg.. EMBO J.

[62] Fleming WH, Hamel A, MacDonald R (1986). Expression of the c-myc protooncogene in human prostatic carcinoma and benign prostatic hyperplasia.. Cancer Res.

[63] Hornberg E, Ylitalo EB, Crnalic S (2011). Expression of androgen receptor splice variants in prostate cancer bone metastases is associated with castration-resistance and short survival.. PLoS One.

[64] Bernard D, Pourtier-Manzanedo A, Gil J, Beach DH (2003). Myc confers androgen-independent prostate cancer cell growth.. J Clin Invest.

[65] Bai S, Cao S, Jin L (2019). A positive role of c-Myc in regulating androgen receptor and its splice variants in prostate cancer.. Oncogene.

[66] Nadiminty N, Tummala R, Liu C (2015). NF-kappaB2/p52:c-Myc:hnRNPA1 pathway regulates expression of androgen receptor splice variants and enzalutamide sensitivity in prostate cancer.. Mol Cancer Ther.

[67] Barfeld SJ, Fazli L, Persson M (2015). Myc-dependent purine biosynthesis affects nucleolar stress and therapy response in prostate cancer.. Oncotarget.

[68] Jiang P, Du W, Yang X (2013). p53 and regulation of tumor metabolism.. J Carcinog.

[69] Zawacka-Pankau J, Grinkevich VV, Hunten S (2011). Inhibition of glycolytic enzymes mediated by pharmacologically activated p53: targeting Warburg effect to fight cancer.. J Biol Chem.

[70] Yahagi N, Shimano H, Matsuzaka T (2003). p53 Activation in adipocytes of obese mice.. J Biol Chem.

[71] Simabuco FM, Morale MG, Pavan ICB (2018). p53 and metabolism: from mechanism to therapeutics.. Oncotarget.

[72] Cronauer MV, Schulz WA, Burchardt T, Ackermann R, Burchardt M (2004). Inhibition of p53 function diminishes androgen receptor-mediated signaling in prostate cancer cell lines.. Oncogene.

[73] Maughan BL, Guedes LB, Boucher K (2018). p53 status in the primary tumor predicts efficacy of subsequent abiraterone and enzalutamide in castration-resistant prostate cancer.. Prostate Cancer Prostatic Dis.

[74] Castro E, Romero-Laorden N, Del Pozo A (2019). PROREPAIR-B: a prospective cohort study of the impact of germline DNA repair mutations on the outcomes of patients with metastatic castration-resistant prostate cancer.. J Clin Oncol.

[75] Nombela P, Lozano R, Aytes A (2019). BRCA2 and other DDR genes in prostate cancer.. Cancers (Basel).

[76] Privat M, Radosevic-Robin N, Aubel C (2014). BRCA1 induces major energetic metabolism reprogramming in breast cancer cell.. PLoS One.

[77] Zadra G, Photopoulos C, Loda M (2013). The fat side of prostate cancer.. Biochim Biophys Acta.

[78] Patel M, Infante J, Von Hoff D (2015). Report of a first-in-human study of the first-in-class fatty acid synthase (FASN) inhibitor TVB-2640.. Cancer Res.

[79] Brenner AJ, Falchook G, Patel M (2017). Abstract P6-11-09: heavily pre-treated breast cancer patients show promising responses in the first in human study of the first-in-class fatty acid synthase (FASN) inhibitor, TVB-2640 in combination with paclitaxel.. Cancer Res.

[80] Ventura R, Mordec K, Waszczuk J (2015). Inhibition of de novo palmitate synthesis by fatty acid synthase induces apoptosis in tumor cells by remodeling cell membranes, inhibiting signaling pathways, and reprogramming gene expression.. EBioMedicine.

[81] Yang H, Pang L, Hu X (2020). The effect of statins on advanced prostate cancer patients with androgen deprivation therapy or abiraterone/enzalutamide: a systematic review and meta-analysis.. J Clin Pharm Ther.

[82] Shah S, Carriveau WJ, Li J (2016). Targeting ACLY sensitizes castration-resistant prostate cancer cells to AR antagonism by impinging on an ACLY-AMPK-AR feedback mechanism.. Oncotarget.

[83] Nassar Z, Centenera M, Machiels J (2019). Lipid elongation in prostate cancer: an androgen regulated process and a novel therapeutic target.. Oncol Abstr.

[84] Tamura K, Makino A, Hullin-Matsuda F (2009). Novel lipogenic enzyme ELOVL7 is involved in prostate cancer growth through saturated long-chain fatty acid metabolism.. Cancer Res.

[85] Iglesias-Gato D, Thysell E, Tyanova S (2018). The proteome of prostate cancer bone metastasis reveals heterogeneity with prognostic implications.. Clin Cancer Res.

[86] Flaig TW, Salzmann-Sullivan M, Su LJ (2017). Lipid catabolism inhibition sensitizes prostate cancer cells to antiandrogen blockade.. Oncotarget.

[87] Xian Z, Liu J, Chen Q (2015). Inhibition of LDHA suppresses tumor progression in prostate cancer.. Tumour Biol.

[88] Muramatsu H, Sumitomo M, Morinaga S (2019). Targeting lactate dehydrogenase-A promotes docetaxel-induced cytotoxicity predominantly in castration-resistant prostate cancer cells.. Oncol Rep.

[89] Xu L, Ma E, Zeng T (2019). ATM deficiency promotes progression of CRPC by enhancing Warburg effect.. Endocr Relat Cancer.

[90] Bushunow P, Reidenberg MM, Wasenko J (1999). Gossypol treatment of recurrent adult malignant gliomas.. J Neurooncol.

[91] Valencia T, Kim JY, Abu-Baker S (2014). Metabolic reprogramming of stromal fibroblasts through p62-mTORC1 signaling promotes inflammation and tumorigenesis.. Cancer Cell.

[92] Mishra R, Haldar S, Placencio V (2018). Stromal epigenetic alterations drive metabolic and neuroendocrine prostate cancer reprogramming.. J Clin Invest.

[93] Ippolito L, Morandi A, Taddei ML (2019). Cancer-associated fibroblasts promote prostate cancer malignancy via metabolic rewiring and mitochondrial transfer.. Oncogene.

[94] Pértega-Gomes N, Vizcaíno José R, Vizcaíno JR (2014). A lactate shuttle system between tumour and stromal cells is associated with poor prognosis in prostate cancer.. BMC Cancer.

[95] Vidal AC, Howard LE, Sun SX (2017). Obesity and prostate cancer-specific mortality after radical prostatectomy: results from the shared equal access regional cancer hospital (SEARCH) database.. Prostate Cancer Prostatic Dis.

[96] Peck B, Schulze A (2019). Lipid metabolism at the nexus of diet and tumor microenvironment.. Trends Cancer.

[97] Labbe DP, Zadra G, Yang M (2019). High-fat diet fuels prostate cancer progression by rewiring the metabolome and amplifying the MYC program.. Nat Commun.

[98] Ngo TH, Barnard RJ, Anton T (2004). Effect of isocaloric low-fat diet on prostate cancer xenograft progression to androgen independence.. Cancer Res.

[99] Lin PH, Aronson W, Freedland SJ (2019). An update of research evidence on nutrition and prostate cancer.. Urol Oncol.

[100] Freedland SJ, Howard L, Allen J (2019). A lifestyle intervention of weight loss via a low-carbohydrate diet plus walking to reduce metabolic disturbances caused by androgen deprivation therapy among prostate cancer patients: carbohydrate and prostate study 1 (CAPS1) randomized controlled trial.. Prostate Cancer Prostatic Dis.

[101] Freedland SJ, Allen J, Jarman A (2020). A Randomized controlled trial of a 6-month low-carbohydrate intervention on disease progression in men with recurrent prostate cancer: carbohydrate and prostate study 2 (CAPS2).. Clin Cancer Res.

[102] Kelly RS, Vander Heiden MG, Giovannucci E, Mucci LA (2016). Metabolomic biomarkers of prostate cancer: prediction, diagnosis, progression, prognosis, and recurrence.. Cancer Epidemiol Biomarkers Prev.

[103] Kdadra M, Hockner S, Leung H, Kremer W, Schiffer E (2019). Metabolomics biomarkers of prostate cancer: a systematic review.. Diagnostics (Basel).

[104] Huang G, Liu X, Jiao L (2014). Metabolomic evaluation of the response to endocrine therapy in patients with prostate cancer.. Eur J Pharmacol.

[105] Lin HM, Mahon KL, Weir JM (2017). A distinct plasma lipid signature associated with poor prognosis in castration-resistant prostate cancer.. Int J Cancer.

[106] Randall EC, Zadra G, Chetta P (2019). Molecular characterization of prostate cancer with associated gleason score using mass spectrometry imaging.. Mol Cancer Res.

[107] Ren S, Shao Y, Zhao X (2016). Integration of metabolomics and transcriptomics reveals major metabolic pathways and potential biomarker involved in prostate cancer.. Mol Cell Proteomics.

[108] Meller S, Meyer HA, Bethan B (2016). Integration of tissue metabolomics, transcriptomics and immunohistochemistry reveals ERG- and gleason score-specific metabolomic alterations in prostate cancer.. Oncotarget.

[109] Alexandrov T (2020). Spatial Metabolomics and Imaging Mass Spectrometry in the Age of Artificial Intelligence.. Annu Rev Biomed Data Sci.

[110] Kurhanewicz J, Vigneron D, Carroll P, Coakley F (2008). Multiparametric magnetic resonance imaging in prostate cancer: present and future.. Curr Opin Urol.

[111] Kobus T, Vos PC, Hambrock T (2012). Prostate cancer aggressiveness: in vivo assessment of MR spectroscopy and diffusion-weighted imaging at 3 T.. Radiology.

[112] Bednarova S, Lindenberg ML, Vinsensia M (2017). Positron emission tomography (PET) in primary prostate cancer staging and risk assessment.. Transl Androl Urol.

[113] Nelson SJ, Kurhanewicz J, Vigneron DB (2013). Metabolic imaging of patients with prostate cancer using hyperpolarized [1-(1)(3)C]pyruvate.. Sci Transl Med.

[114] Chen HY, Aggarwal R, Bok RA (2020). Hyperpolarized (13)C-pyruvate MRI detects real-time metabolic flux in prostate cancer metastases to bone and liver: a clinical feasibility study.. Prostate Cancer Prostatic Dis.

[115] Aggarwal R, Vigneron DB, Kurhanewicz J (2017). Hyperpolarized 1-[(13)C]-pyruvate magnetic resonance imaging detects an early metabolic response to androgen ablation therapy in prostate cancer.. Eur Urol.

[116] Dost RJ, Glaudemans AW, Breeuwsma AJ, de Jong IJ (2013). Influence of androgen deprivation therapy on choline PET/CT in recurrent prostate cancer.. Eur J Nucl Med Mol Imaging.

[117] Galgano SJ, Valentin R, McConathy J (2018). Role of PET imaging for biochemical recurrence following primary treatment for prostate cancer.. Transl Androl Urol.

